# Pain and reward circuits antagonistically modulate alcohol expectancy to regulate drinking

**DOI:** 10.1038/s41398-020-00909-z

**Published:** 2020-07-07

**Authors:** Thang M. Le, Simon Zhornitsky, Sheng Zhang, Chiang-Shan R. Li

**Affiliations:** 1 Department of Psychiatry, Yale University School of Medicine, New Haven, CT 06519 USA; 2 Department of Neuroscience, Yale University School of Medicine, New Haven, CT 06520 USA; 3 Interdepartmental Neuroscience Program, Yale University School of Medicine, New Haven, CT 06520 USA

**Keywords:** Neuroscience, Diagnostic markers

## Abstract

Expectancy of physical and social pleasure (PSP) promotes excessive drinking despite the potential aversive effects of misuse, suggesting an imbalance in the response to reward and pain in alcohol seeking. Here, we investigated the competing roles of the reward and pain circuits in PSP expectancy and problem drinking in humans. Using fMRI data during resting (*n* = 180) and during alcohol cue exposure (*n* = 71), we examined the antagonistic effects of the reward-related medial orbitofrontal cortex (mOFC) and pain-related periaqueductal gray (PAG) connectivities on PSP expectancy and drinking severity. The two regions’ connectivity maps and strengths were characterized to assess their shared substrates and net relationship with PSP expectancy. We evaluated mediation and path models to further delineate how mOFC and PAG connectivities interacted through the shared substrates to differentially impact expectancy and alcohol use. During resting, whole-brain regressions showed mOFC connectivity in positive and PAG connectivity in negative association with PSP scores, with convergence in the precentral gyrus (PrCG). Notably, greater PAG-PrCG relative to mOFC-PrCG connectivity strength predicted lower PSP expectancy. During the alcohol cue exposure task, the net strength of the PAG vs. mOFC cue-elicited connectivity with the occipital cortex again negatively predicted PSP expectancy. Finally, mediation and path models revealed that the PAG and mOFC connectivities indirectly and antagonistically modulated problem drinking via their opposing influences on expectancy and craving. Thus, the pain and reward circuits exhibit functional antagonism such that the mOFC connectivity increases expectancy of drinking pleasure whereas the PAG serves to counter that effect.

## Introduction

Alcohol expectancy is a powerful determinant of drinking behavior^1^. Anticipation of rewarding alcohol effects, particularly physical and social pleasure (PSP), has been shown to reliably predict immediate consumption and future misuse^2,3^. Expectancy of drinking pleasure, however, is lessened by anticipated negative consequences (e.g., hangover, impaired judgment, etc.) which can serve to prevent excessive intake and escalation to dependence^4,5^. Thus, alcohol expectancy is regulated by the balance between two opposing responses to drinking. When biased towards PSP, alcohol expectancy represents a significant risk factor for abuse, as posited by motivational theories of alcohol use^6,7^.

The involvement of positive and negative alcohol effects in determining expectancy of PSP suggests the recruitment of both reward and pain circuits. Indeed, regions associated with reward processing, especially the medial orbitofrontal cortex (mOFC), have been consistently implicated in both the positive perception and pleasure of drinking. Alcohol administration induced endogenous opioid release in the mOFC^8^ and the feeling of pleasure^9^. The mOFC also increases activation to alcohol ingestion and cues^10,11^ and this activity was predictive of craving ratings in alcoholics^12^. In contrast, the aversive effects of alcohol elicit pain responses and activate the periaqueductal gray (PAG) of the pain circuit which in turn may play a role in reducing future consumption. Indeed, disruptions of PAG-medial prefrontal cortex connections in mice led to compulsive drinking despite the pairing of foot shock and unpleasant taste with alcohol^13^. This finding demonstrates the critical role of the PAG in the negative response to drinking. Excessive ethanol intake increased neuronal activity and altered expressions of anxiety-related genes in the PAG^14,15^, consistent with reports of alcohol withdrawal-induced anxiety and hyperalgesia in humans^16^ and rodents^17,18^. Together, such evidence establishes the important function of the mOFC and PAG in assessing the positive and negative effects of alcohol. As reward and pain antagonistically influence alcohol expectancy, their underlying brain circuits may also compete to modulate drinking behavior. Nevertheless, how they interact to regulate expectancy of PSP and alcohol misuse remains unclear.

While brain regions implicated in pain and reward processing play a crucial role in the response to motivationally significant stimuli, they likely work in conjunction with other structures to guide behaviors. For instance, alcohol administration was shown to enhance functional connectivity of multiple domains including the affective^19^, visual^20^, motor^21^, and frontal^22^ cortices. During noxious thermal stimulation, the dorsolateral prefrontal cortical connectivity with the mOFC increased with heat while its connectivity with the PAG positively predicted opioid release in the PAG during placebo treatment^23^, potentially reflecting the mechanisms underlying expected pain and pain relief. Thus, it is plausible that the sensory and frontal systems serve as the common substrates for the interaction of reward and pain circuits during the regulation of anticipation, perception, and behavior. Whether such interactions are present in alcohol expectancy or how they may dictate drinking behavior is poorly understood.

Here, we characterized the pain and reward circuits’ antagonistic connectivities in relation to expectancy of PSP and quantify their differential effects on drinking severity. As alcohol expectancy represents a stable psychological construct but one which can be activated or enhanced in response to external cues^24^, the relationship between expectancy of PSP and brain connectivities was examined during both resting state and during alcohol cue exposure. During resting, we tested the hypothesis that mOFC functional connectivity would be associated with greater expectancy of PSP whereas PAG connectivity would exhibit the opposite pattern. As the two circuits likely showed mutual antagonism, we expected their net connectivity strength to determine the degree of expectancy. We next confirmed the presence of such antagonism during alcohol cue exposure. Finally, models in which the mOFC and PAG interacted to regulate alcohol expectancy, craving, and misuse were evaluated. Together, our findings shed light on how the competing mOFC and PAG impact alcohol misuse via the expectancy of drinking pleasure.

## Methods

### Participants

One hundred and eighty adult drinkers (85 females; age = 37.7 ± 13.9 years) participated in the study. Subjects provided written informed consent after details of the study were explained in accordance to institute guidelines approved by the Yale Human Investigation Committee.

Participants completed the PSP subscale of the Alcohol Expectancy Questionnaire^25^ (Supplemental Methods), reporting a PSP score of 19.6 ± 5.9 (mean ± SD) with higher scores indicating greater expectancy. Participants also completed the Alcohol Use Disorder Identification Test (AUDIT)^26^ with higher scores suggesting greater risk for having or developing an alcohol use disorder. An average AUDIT score of 6.8 ± 7.4 indicated moderate severity of problem drinking^27^. The 71 participants who performed the alcohol cue craving task further reported their drinking duration (in years) and alcohol craving. In addition, a self-assessment measuring out-of-control drinking behavior on a scale from 0 to 10 (0 = completely in control, 10 = completely out of control) was administered.

### Resting and alcohol cue reactivity task

All 180 subjects completed one session of 10-min resting-state fMRI. Following the resting-state run, a subsample of 71 subjects (34 females, age = 36.1 ± 14.0 years) also performed an alcohol cue reactivity (ACR) task in the same fMRI session. The remaining subjects were part of a different study.

In the ACR task (Fig. S1), participants viewed alternating blocks of alcohol-related (e.g., alcoholic drinks, bar scenes, etc.) and neutral (e.g., milk, orange juice, etc.) images. In each block, after a 2-s fixation, six pictures displaying alcohol (alcohol block) or neutral (neutral block) cues were shown for 6 s each. Participants were instructed to view the stimuli and contemplate how they may relate to them. At the end of each block, participants rated their alcohol craving on a scale of 0–10 (0 = no craving at all, 10 = highest craving possible). Participants completed two 9-min runs with each run consisting of six alcohol and six neutral blocks.

### Imaging protocol

Conventional T1-weighted spin echo sagittal anatomical images were acquired for slice localization using a 3T scanner (Siemens Trio). Anatomical images of the functional slice locations were next obtained with spin echo imaging in the axial plane parallel to the AC–PC line with (TR) = 1900 ms, echo time (TE) = 2.52 ms, bandwidth = 170 Hz/pixel, FOV = 250 × 250 mm, matrix = 256 × 256, 176 slices with slice thickness = 1 mm and no gap. Functional blood oxygenation level-dependent (BOLD) signals were acquired using multiband imaging (multiband acceleration factor = 3) with a single-shot gradient echo echoplanar imaging sequence. Fifty-one axial slices parallel to the AC–PC line covering the whole brain were acquired with TR = 1000 ms, TE = 30 ms, bandwidth = 2290 Hz/pixel, flip angle = 62◦, field of view = 210 × 210 mm, matrix = 84 × 84, with slice thickness = 2.5 mm and no gap. Data preprocessing steps follow our previous work^28^ and are reported in Supplementary Methods.

### Functional connectivity–resting state

For seed-based resting-state functional connectivity (rsFC), we employed masks of the mOFC (MNI coordinates *x* = 2, *y* = 46, *z* = −8, *k* = 146) obtained from an imaging meta-analysis examining reward processing^29^ and the PAG (*x* = 0, *y* = −32, *z* = −9, *k* = 30) from the Harvard Ascending Arousal Network^30^ (Fig. S2). The correlation coefficient between the averaged time course of the seed region and that of every other voxel was computed then Fisher’s z transformed for each subject. The Z maps were used in group, random effect analyses in which we conducted whole-brain multiple regressions against the PSP scores, with age and sex as the covariates. All imaging results were examined with the threshold of voxel-level *p* < 0.001 (uncorrected) in combination with cluster-level *p* < 0.05 (corrected for family-wise error).

### Cue-elicited activations and connectivities—ACR task

A general linear model (GLM) was constructed as in previous work with similar task designs^31,32^ to differentiate regional activations to alcohol and neutral cues (Supplemental Methods). One-sample *t*-tests were conducted at the individual subject level to evaluate alcohol vs. neutral cue contrasts.

Cue-elicited connectivity of the PAG and mOFC during alcohol vs. neutral cues was estimated using general psychophysiological interactions (gPPI)^33^ (Supplemental Methods). We used the same PAG and mOFC seeds as in the rsFC analysis. As the occipital cortex (OC) showed preferential responses to alcohol cues (see “Results”), we examined its cue-elicited connectivity with the PAG and mOFC in a region-of-interest analysis. We extracted parameter estimates which represented their connectivity strength for the contrast alcohol >neutral cues and assessed their relationship with PSP scores, AUDIT scores, and alcohol craving ratings.

### Mediation and path analyses

To examine the inter-relationships of PAG, mOFC connectivities, PSP, and AUDIT scores, we conducted mediation analyses^34^ (Supplemental Methods). For each seed, we considered all six models, with each model featuring the following as the independent variable (*X*), dependent variable (*Y*), and mediator (*M*), respectively: Model 1—functional connectivity, AUDIT scores, and PSP scores; Model 2—AUDIT, connectivity, and PSP; Model 3—AUDIT, PSP, and connectivity; Model 4—PSP, AUDIT, and connectivity; Model 5—connectivity, PSP, and AUDIT; Model 6—PSP, connectivity, and AUDIT.

We also employed path analysis to evaluate how PAG and mOFC connectivity with the precentral gyrus (denoted as PAG/PrCG and mOFC/PrCG from here on) during resting state (see “Results”) as well as with the OC during the ACR task modulated PSP and AUDIT scores. Model fit was assessed with standard fit indices^35,36^ (Supplemental Methods). For resting-state data, we evaluated the model in which PAG and mOFC connectivities differentially influenced alcohol expectancy which in turn affected problem drinking. An alternative model in which PSP scores influenced AUDIT scores which in turn modulated mOFC/PrCG and PAG/PrCG connectivities was also considered. For alcohol cue-elicited data, we evaluated the model in which difference in connectivity strength of the PAG and mOFC indirectly modulated problem drinking by influencing alcohol expectancy and craving. An alternative model in which PSP indirectly influenced connectivity difference via AUDIT and craving was also considered. Both direct and indirect effects were examined with bootstrapping^37^ to determine how connectivities from the pain and reward circuits modulated problem drinking and whether these modulations were subjected to mediation effects.

## Results

### Drinking behavior assessments

All 180 participants reported PSP scores (M ± SD = 19.6 ± 5.9) and AUDIT scores (6.8 ± 7.4), with higher scores indicating greater expectancy and drinking severity. During the ACR task, 71 participants further rated their alcohol craving (3.2 ± 2.7). The self-assessment for out-of-control of the same group yielded an average score of 1.7 ± 2.4. As expected, there were significant positive relationships between the scores of PSP, AUDIT, alcohol craving, and out-of-control drinking assessment (*p*’s < 0.01, Table S1).

### Alcohol expectancy and PAG resting-state connectivity

**Fig. 1:**
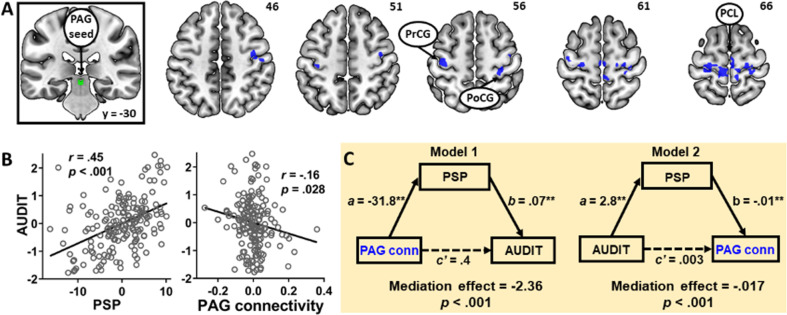
PAG connectivity and alcohol expectancy. **a** Whole-brain multiple regression showed a negative correlation between PSP and PAG (far left in green) connectivity with the precentral gyrus (PrCG), postcentral gyrus (PoCG), and paracentral lobule (PCL) (right, blue). **b** AUDIT scores were positively correlated with PSP scores (left) and negatively correlated with averaged connectivity strength of PAG with PrCG, PoCG, and PCL (right). **c** Mediation analysis showed two significant models in which PSP bidirectionally mediated the negative relationship between PAG connectivity and AUDIT scores. NB: all partial correlations in this and other figures show regression residuals of parameter estimates of connectivity, PSP, and AUDIT scores after the effects of age and sex had been removed. **p < 0.01.

For rsFC, we used the PAG as the seed and performed a whole-brain multiple regression of PAG connectivity using PSP scores as the predictor. Results showed a negative correlation between PSP scores and PAG connectivity with the bilateral precentral gyrus (PrCG), right postcentral gyrus (PoCG), and paracentral lobule (PCL) (Fig. 1a, Table S2). No clusters showed a significant positive correlation between PSP scores and PAG connectivity.

As PSP scores were negatively correlated with PAG rsFC and both showed a significant relationship with AUDIT scores (Fig. 1b), we used mediation analysis to examine their inter-relationships. PAG rsFC was calculated by averaging the parameter estimates of PAG connectivity with PrCG, PoCG, and PCL as identified by the multiple regression. The results showed two significant models (Fig. 1c, Table S3). In Model 1, PAG connectivity reduced PSP expectancy, which in turn lowered problem drinking: PAG connectivity → PSP → AUDIT. The model showed a significant and full mediation effect after correction for multiple model testing (*c* − *c*’ = −2.36, *p* < 0.001, Supplemental Results). Model 2 (AUDIT → PSP → PAG connectivity) also showed a significant and full mediation effect (*c* − *c*’ = −0.017, *p* < 0.001). None of the other four models was significant (corrected *p*’s > 0.06). Taken together, enhanced PAG connectivity with the PrCG, PoCG, and PCL was associated with lower drinking severity and this negative relationship was fully and bidirectionally mediated by reduced PSP expectancy.

### Alcohol expectancy and mOFC resting-state connectivity

**Fig. 2:**
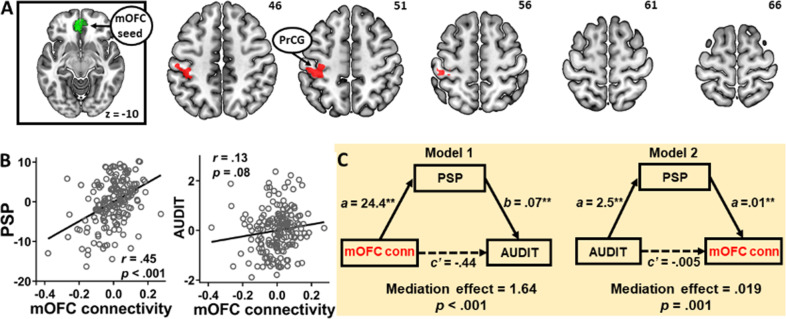
Medial OFC connectivity and alcohol expectancy. **a** Whole-brain multiple regression showed positive correlation between PSP scores and the mOFC (far left in green) connectivity with the left precentral gyrus (PrCG) (right, red). **b** mOFC/PrCG connectivity strength showed a significant positive correlation with PSP (left) and a near significant positive correlation with AUDIT scores (right). **c** Mediation analysis showed two significant models in which PSP bidirectionally mediated the positive relationship between mOFC/PrCG connectivity and AUDIT scores. Note: mOFC/PrCG denotes mOFC connectivity with PrCG. **p < 0.01.

Using the mOFC as the seed, we performed a whole-brain multiple regression of the mOFC rsFC with PSP scores as the predictor. Results showed a positive correlation between PSP scores and mOFC connectivity with the left PrCG (Fig. 2a, Table S2). No significant negative correlation for mOFC connectivity and PSP scores was found.

As with the PAG connectivity, we examined the inter-relationship between mOFC/PrCG connectivity, PSP, and AUDIT scores in a mediation analysis. Medial OFC/PrCG connectivity showed a significant positive correlation with PSP as expected (Fig. 2b left) and a near significant correlation with AUDIT scores (*p* = 0.08, Fig. 2b right). There was a significant mediation effect in Model 1 (mOFC connectivity → PSP → AUDIT) after correction for multiple model testing (*c* − *c*’ = 1.64, *p* < 0.001) (Fig. 2c, Table S4). However, while path coefficient *c* was significantly weakened after accounting for the mediating effect of PSP, the relationship between mOFC connectivity and AUDIT scores only reached near significance (*p* = 0.10). It is worth noting that the significance of this relationship is not considered a requirement for mediation analysis^38^. Model 2 (AUDIT → PSP → mOFC connectivity) also showed a significant mediation effect (*c* − *c*’ = 0.019, *p* = 0.001). Again, the relationship between mOFC connectivity and AUDIT scores was only trending toward significance (*p* = 0.07). None of the remaining models was significant (*p*’s > 0.50). Thus, elevated mOFC connectivity with the PrCG was associated with increased drinking severity and this relationship was bidirectionally mediated by heightened PSP expectancy.

### Opposing effects of PAG and mOFC rsFC on alcohol expectancy and misuse

**Fig. 3:**
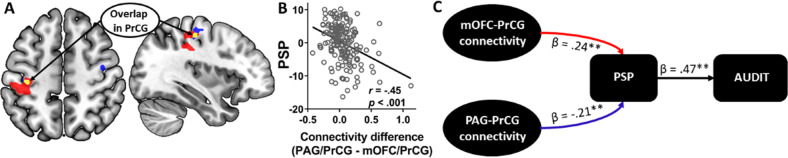
Functional antagonism of PAG and mOFC rsFC. **a** PSP scores were negatively correlated with PAG rsFC (blue) but positively correlated with mOFC rsFC (red) and the two connectivity maps overlapped in the left PrCG (yellow). **b** PSP scores were negatively predicted by the difference in connectivity strength between PAG/PrCG and mOFC/PrCG, indicating the stronger the PAG/PrCG relative to mOFC/PrCG connectivity, the lower PSP expectancy. **c** Path analysis showed PAG and mOFC rsFC with the PrCG indirectly and antagonistically influenced drinking severity through their modulation of expectancy. PAG/PrCG rsFC lessened drinking severity by reducing PSP expectancy (blue arrow). In contrast, mOFC/PrCG rsFC exacerbated drinking severity by enhancing PSP expectancy (red arrow). *β*’s represent path coefficients. **p < 0.01.

To determine whether the rsFC patterns of the PAG and mOFC shared common substrates, we examined the PAG and mOFC connectivity maps which overlapped in the left PrCG (Fig. 3a). Thus, mOFC/PrCG connectivity exhibited a positive relationship with PSP scores whereas PAG/PrCG connectivity showed a negative relationship (Fig. S3).

To quantify the opposing effects of PAG and mOFC rsFC on alcohol expectancy, we first calculated the difference in strength of their connectivity with the overlapping PrCG (i.e., [PAG connectivity with PrCG] minus [mOFC connectivity with PrCG]). This net connectivity strength significantly and negatively predicted PSP scores (*r* = −0.45, *p* < 0.001, Fig. 3b). Thus, the stronger the PAG/PrCG relative to mOFC/PrCG connectivity, the lower the PSP expectancy.

We next employed path analysis to further characterize the antagonism of PAG and mOFC rsFC with the PrCG in modulating expectancy and drinking severity in the same model. We tested the scenario in which PAG and mOFC connectivities indirectly influenced AUDIT scores via their opposing effects on PSP expectancy (Fig. 3c). Parameter estimates of the PAG and mOFC rsFC with the PrCG were used as the two exogenous variables. The model showed a good fit (Fit indices: RMSEA = 0.00 [90% CI: 0.00 0.11], *χ*^2^/df = 0.33, SRMR = 0.02, and CFI = 1.00). PSP scores were negatively modulated by PAG/PrCG connectivity (*β* = −0.21, *p* = 0.004) but positively modulated by mOFC/PrCG connectivity (*β* = 0.24, *p* = 0.001). Bootstrapping assessing indirect effects showed significant mediation effects of PSP on the relationship of AUDIT scores with PAG as well as with mOFC connectivities. Specifically, PAG/PrCG connectivity decreased problem drinking through reducing PSP expectancy (*β* = −0.12, *p* = 0.001, full mediation). Conversely, mOFC/PrCG connectivity increased problem drinking through enhancing PSP expectancy (*β* = 0.11, *p* = 0.004, full mediation). Thus, problem drinking was positively modulated by the connectivity of the reward circuit but negatively modulated by that of the pain circuit and these modulations were achieved indirectly via PSP expectancy.

We also considered the model in which PSP scores differentially modulated PAG and mOFC connectivity via AUDIT scores (Fig. S4). The model showed a good fit (Fit indices: RMSEA = 0.00, *χ*^2^/df = 0.245, SRMR = 0.333, and CFI = 1.00), indicating the direction of influence between PAG as well as mOFC connectivity and expectancy may be bidirectional.

The alternative model in which PSP scores influenced AUDIT scores which in turn modulated PAG and mOFC connectivities showed a poor fit (Fit indices: RMSEA = 0.23, *χ*^2^/df = 10.80, SRMR = 0.10, and CFI = 0.73, Fig. S4) and was not considered further.

### Cue-related PAG and mOFC connectivities, alcohol expectancy, and problem drinking

**Fig. 4:**
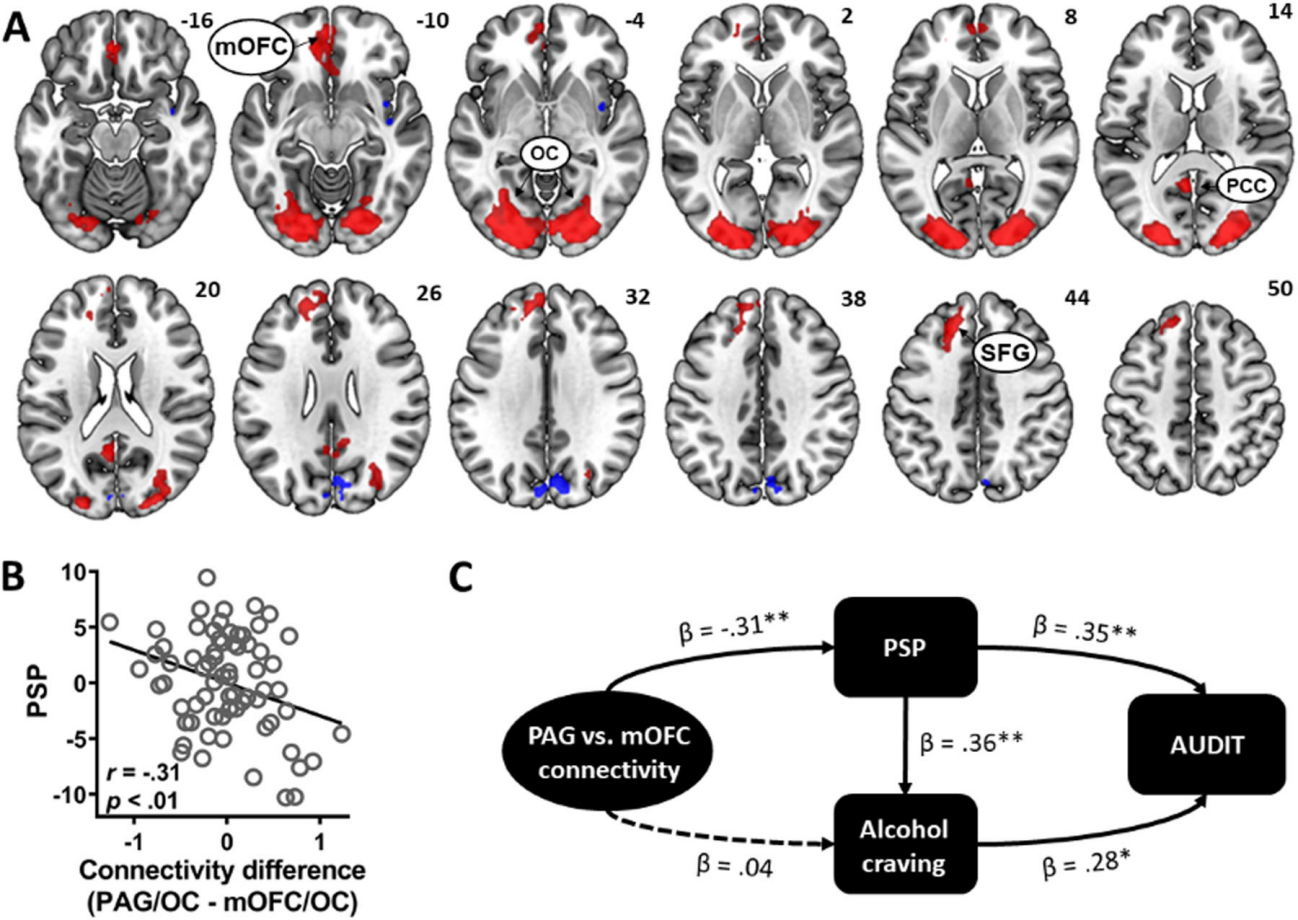
Functional antagonism of PAG and mOFC connectivity during alcohol cue exposure. **a** During the ACR task, alcohol relative to neutral cues elicited activations in the mOFC, superior frontal gyrus (SFG), and a cluster containing the bilateral occipital cortices (OC) and posterior cingulate cortex (PCC) (red). The reverse contrast showed significant activation in the parieto-occipital sulcus (blue). **b** PSP scores were negatively correlated with the difference in connectivity strength between PAG/OC and mOFC/OC during alcohol cue processing, indicating the stronger the PAG/OC relative to mOFC/OC connectivity, the lower the PSP expectancy. **c** Path analysis showed a significant model in which PAG vs. mOFC connectivity strength difference indirectly reduced problem drinking by lowering PSP, which in turn lowered alcohol craving. Solid and dotted lines show significant and non-significant paths, respectively. *p < 0.05, **p < 0.01.

For the ACR task, we identified the brain regions with preferential responses to alcohol cues. The contrast alcohol > neutral cues showed significant activations in the mOFC, bilateral OC, posterior cingulate cortex, and left superior frontal gyrus (Fig. 4a, Table S5). The reverse contrast neutral >alcohol cues yielded significant activation in the parieto-occipital sulcus.

As the OC is shown here and elsewhere^10^ to exhibit robust responses to alcohol cues, we next determined whether the cue-elicited connectivity of the OC with the PAG and mOFC modulated PSP expectancy during alcohol cue exposure. Here, the OC was defined from the alcohol > neutral cue contrast. PPI model was estimated with the PAG and mOFC as the seeds for the connectivity with the OC during alcohol vs. neutral cues. Parameter estimates of the PAG connectivity with OC and mOFC connectivity with OC were then extracted for the gPPI contrast alcohol > neutral cues. We then examined their relative strength in relation to alcohol expectancy. The difference in connectivity strength of the PAG and mOFC with the OC (i.e., [PAG connectivity with OC] minus [mOFC connectivity with OC]) was significantly and negatively correlated with PSP scores (*r* = −0.31, *p* < 0.01, Fig. 4b). Thus, stronger PAG connectivity strength relative to the mOFC connectivity, both with the OC, predicted lower PSP expectancy during alcohol cue exposure. As with the rsFC, the finding indicates competing roles of PAG and mOFC connectivities in modulating alcohol expectancy.

Alcohol craving ratings showed a significant relationship with both PSP (*r* = 0.34, *p* = 0.004) and AUDIT (*r* = 0.38, *p* = 0.001) scores. Thus, we next examined how the difference in connectivity strength between PAG/OC and mOFC/OC may influence alcohol expectancy, craving, and misuse in a path analysis (Fig. 4c). We tested the model in which PAG vs. mOFC connectivity strength difference indirectly influenced AUDIT scores by negatively modulating PSP and alcohol craving. The model showed a good fit (Fit indices: RMSEA = 0.00 [90% CI: 0.00 0.24], *χ*^2^/df = 0.17, SRMR = 0.013, and CFI = 1.00). Specifically, PAG vs. mOFC connectivity strength difference significantly reduced PSP scores (*β* = −0.31, *p* = 0.006) which in turn affected alcohol craving (*β* = 0.36, *p* = 0.002). Both PSP and craving rating then increased AUDIT scores (*p*’s ≤ 0.011). Thus, PAG relative to mOFC connectivity lowered PSP expectancy, leading to attenuated alcohol craving, and subsequently decreased problem drinking.

We also considered the model in which PSP modulated PAG vs. mOFC connectivity difference via AUDIT scores (Fig. S5). Results showed that the model showed a good fit (Fit indices: RMSEA = 0.00, *χ*^2^/df = 0.245, SRMR = 0.015, and CFI = 1.00), indicating the direction of influence between PAG vs. mOFC connectivity and expectancy of drinking pleasure may be bidirectional.

The alternative model in which PSP expectancy indirectly influenced PAG/mOFC connectivity difference via problem drinking and craving showed a poor fit (Fit indices: RMSEA = 0.29, *χ*^2^/df = 6.92, SRMR = 0.08, and CFI = 0.81, Fig. S5) and was not considered further. Finally, defining the OC using a more stringent threshold (Fig. S6) did not materially change the results.

## Discussion

We identified the competing modulation of the reward and pain circuits on alcohol expectancy by characterizing functional connectivities of the mOFC and PAG during resting state and during alcohol cue exposure. Our findings were threefold. First, elevated rsFC of the mOFC was associated with heightened expectancy of drinking pleasure which in turn predicted greater drinking severity. In contrast, PAG connectivity exhibited the opposite influence. Second, the mOFC and PAG rsFC maps converged in the precentral gyrus (PrCG). Through the PrCG, the mOFC and PAG indirectly and antagonistically modulated drinking behavior by exerting opposing effects on alcohol expectancy. Further, their net connectivity strength predicted the degree of expectancy. Finally, during alcohol cue exposure, the PAG and mOFC cue-elicited connectivities, both involving the same visual area, again exhibited competing modulation on alcohol expectancy and drinking severity. Together, these findings support the functional antagonism between the pain and reward circuits such that PAG connectivity protects against problem drinking while mOFC connectivity serves as a risk factor for alcohol misuse.

The PAG plays a central role in nociception, responding to both anticipated^39^ and perceived^40^ pain. In problem drinking, the PAG has been implicated in hyperalgesia^41–43^. Alcohol dependence frequently produces sustained negative emotional states and chronic pain possibly through the alcohol-induced alterations of the brain mechanisms supporting stress response and pain transmission, both of which involve the PAG^44^. Structural abnormalities of the PAG in dependent individuals were previously observed^45^, corroborating the reports of dysphoria and increased pain sensitivity in this population^46^. In rodents, alcohol administration and binge drinking not only elevated neuronal activity^47^ but also induced changes in N-methyl-D-aspartate and serotonin receptor gene expressions in the PAG, likely increasing susceptibility to pain, fear, and anxiety^15^. In addition, alcohol withdrawal in rats raised concentrations of pain signal-related nitric oxide in the PAG^14^. Collectively, previous evidence suggests the PAG responds to the aversive effects of alcohol use, thus countering the expectancy of drinking pleasure.

The PAG interacts with other brain structures to regulate motivated behaviors^48^ including the motor/premotor cortex which is anatomically connected with the PAG^49^ and responsive to both actual and anticipated pain^50,51^. In particular, the connectivity between the PAG and the PrCG, PoCG, and PCL was shown to increase with thermal pain intensity^52^, mirroring the current rsFC finding of the PAG. It is plausible that the negative modulation of PAG connectivity on pleasure expectancy and problem drinking was reflective of the PAG’s response to anticipated aversive consequences, leading to the suppression of alcohol-seeking behavior. To our knowledge, our imaging evidence is the first to substantiate the role of the pain circuit in deterring problem drinking in humans.

While alcohol misuse can produce painful effects, many individuals continue to drink to excess, reflecting a bias towards the anticipated pleasure derived from drinking. The mOFC has been implicated in supporting such rewarding effects of alcohol and other substances^53^. Alcohol odor and visual cues elicit activation of the mOFC both in healthy and dependent individuals^54,55^ and this mOFC activity positively predicts subsequent relapse in the latter group^56^. Reduced mOFC rsFC with the amygdala, a subcortical hub of the saliency circuit, has been associated with lower alcohol consumption in adolescence^57^. The mOFC, therefore, is involved not only in the pleasant sensation of alcohol consumption but also in the positive perception of drinking, consistent with the current work associating mOFC connectivity with heightened expectancy of pleasure.

The opposite relationships of PAG and mOFC connectivities with alcohol expectancy indicate antagonistic influences of the reward and pain circuits. Past behavioral investigations have underlined the competing roles of expectations for aversive and rewarding outcomes in alcohol consumption. Anticipated negative consequences discourage whereas anticipated pleasure facilitates alcohol use^1,2,58^. As such, their relative strengths determine drinkers’ net expectancy of drinking pleasure and subsequent alcohol-seeking behavior^59^. One likely engages in alcohol use if the perceived benefits from drinking outweighs the perceived costs. While the competing nature of pain and pleasure in alcohol use has long been acknowledged^60^, the neural basis underlying such competition was poorly understood. Our finding that the difference in connectivity strength of the two systems predicted the degree of pleasure expectancy represents new and robust evidence delineating how the reward and pain circuits interact to regulate drinking.

During resting state, the functional antagonism of the reward and pain circuits involved the PrCG which may serve as their shared target to influence drinking behavior. In alcohol dependent individuals, the PrCG exhibited elevated activation to alcohol cues and craving ratings^61,62^ and this activation was reduced following cue-exposure extinction treatment^63^. A more direct relationship between PrCG and alcohol expectancy was demonstrated by the association of increased gray matter volume and event-related potentials of the PrCG with heightened positive alcohol expectancy^64,65^. In contrast, PrCG activity has also been associated with risk avoidance and reduced problem drinking^66^, indicating the region’s complex role in alcohol use. Dictating motor outputs, the PrCG may enable behavioral responses to motivationally significant events^67^. Thus, signals from the mOFC and PAG likely help drive PrCG in facilitating approach or avoidance in alcohol seeking, respectively.

During alcohol cue exposure, we again observed the opposing effects of the PAG and mOFC but with the involvement of the OC. While the recruitment of the visual cortex may not be surprising given its robust activation to alcohol cues^10^ and anatomical connections with both mOFC^68^ and PAG^69^, the current findings suggest a more direct role of the OC in shaping drinking behavior. Previous theoretical work proposes that the visual cortex links perceptual processing of drug cues to the psychophysiological responses to actual consumption^70^. Accordingly, the interaction of the OC with the motivational circuits helps enhance the incentive salience of alcohol cues. In the current study, OC/PAG and OC/mOFC cue-elicited connectivity potentially signaled the negative and positive values of alcohol use, respectively. Their net strength, as a result, likely determines the prevailing affective response that underlines alcohol expectancy. Our path analysis additionally showed the modulations of the two circuits on alcohol craving and expectancy which in turns impacted problem drinking. Such evidence suggests a potential pathway connecting affective processing, perception, craving, and alcohol consumption. Taken together, the current work supports the antagonistic effects of the reward and pain circuits and delineates the involvement of the motor and visual cortices in titrating the expected gains and costs of drinking to regulate alcohol consumption.

Motivational models of alcohol use propose the role of expectancy as a mediator of various risk factors and problem drinking^6,7^. Corroborating evidence shows that trait anxiety^71^, sensation-seeking^72^, and family history of alcoholism^73^ all impact expectancy which in turn predicts alcohol use. As such, alcohol expectancy represents a common pathway through which distal determinants exert influences on drinking behavior^74^. Our results from path and mediation analyses support this conceptual framework. Furthermore, the evidence that the PAG plays a mitigating role whereas the mOFC plays a facilitating one in relation to alcohol misuse extends current understanding of risk factors. Individual differences in connectivity strength of the reward relative to pain systems are shown here to potentially be an additional measure of biological vulnerability for problem drinking.

It is worth noting that our models also suggested that expectancy of drinking pleasure can influence PAG and mOFC connectivities, thus making the relationship between expectancy and reward/pain circuits bidirectional. Specifically, expected pleasure can enhance the reward response to alcohol use and dampen the pain circuit. While such relationship requires further investigation, this interpretation is in line with the notion that substance abuse, including alcohol, can lead to the phenomenon in which drugs “hijack” the brain’s reward system^75^. Problem drinkers seeking stimulation may become increasingly driven by alcohol rewarding effects, even in the face of negative consequences, thus ensuring that the imbalance between pleasure and pain response traps drinkers in a vicious circle.

## Conclusions

Drinking, as with other motivated behaviors, is regulated by the expected gains and costs. Motivational models of alcohol use posit opposite impacts of alcohol expectancies on drinking. Expecting rewarding effects of alcohol promotes alcohol use whereas expecting adverse consequences deters individuals from drinking. Here, we demonstrate how the pain and reward circuit connectivities modulate problem drinking via mutually antagonistic influences on alcohol expectancy. These findings suggest a neural basis of the alcohol expectancy which can serve as markers to predict drinking trajectory and inform treatment designs.

## Supplementary information

Supplementary information
