# Supplementary material for: Pain and reward circuits antagonistically modulate alcohol expectancy to regulate drinking
Source: Transl Psychiatry. 2020 Jul 7;10:220. doi: 10.1038/s41398-020-00909-z (PMC7341762; doi:10.1038/s41398-020-00909-z)
Supplement: Supplementary file 1 — Supplementary information [file 41398_2020_909_MOESM1_ESM.docx]

**Le et al., Pain and reward circuits antagonistically modulate alcohol expectancy to regulate drinking**

***SUPPLEMENTAL INFORMATION***

**1. SUPPLEMENTAL METHODS**

**1.1 Participants**

One hundred and eighty adult drinkers (85 females; age mean ± SD = 37.7 ± 13.9 years, range = 21-75) participated in the study. All subjects were screened to be free from major medical, including neurological, illnesses and lifetime Axis I psychiatric disorders according to DSM-IV. No participants were currently on psychotropic medications and all tested negative for illicit substances on the study day.

Participants completed the Alcohol Expectancy Questionnaire^1^ which includes 40 items measuring 8 subscales of alcohol expectancy. We focused on the Physical and Social Pleasure (PSP) subscale which consists of 5 items assessing the anticipated positive physical and social effects of alcohol. Participants reported an average PSP score of 19.6 ± 5.9 with higher scores indicating greater expectancy. We did not use the remaining subscales of the Alcohol Expectancy Questionnaire as they do not specifically measure the pain or pleasure aspects of drinking. Participants also completed the Alcohol Use Disorder Identification Test (AUDIT)^2^. AUDIT scores are calculated from the sum of 10 self-report questions concerning quantity of alcohol use, alcohol-related problems, adverse reactions, and drinking behavior. A higher score suggests greater risk for having or developing an alcohol use disorder. Participants reported an average AUDIT score of 6.8 ± 7.4, indicating moderate drinking severity and significant inter-subject variability. Age showed a significant negative correlation with PSP (*r* = -.23, *p* = .002) but not AUDIT (*p* = .10) scores. Women showed marginally greater PSP scores than men (*p* = .05) but not drinking severity (*p* = .64). In women, age showed a significant relationship AUDIT (*r* = -.31, *p* = .004) but not PSP (*p* = .07) scores. In men, age was significantly correlated with both AUDIT (*r* = -.28, *p* = .007) and PSP (*r* = -.22, *p* = .03) scores. Participants also reported their drinking duration (in years) as well as a self-assessment of whether they considered their drinking behavior to be out of control on a scale from 0 to 10 (0 = completely in control, 10 = completely out of control). Finally, during the alcohol cue reactivity task (see below), participants rated their craving on a scale of 0 to 10 (0 = no craving at all, 10 = highest craving possible). Correlational results of drinking measures, after controlling for age and sex, are depicted in **Table S1**.

| **Table S1.** Relationships across drinking behavior measures | | | |  |  |
| --- | --- | --- | --- | --- | --- |
|  | PSP | AUDIT | Craving rating**^** | Years of drinking | Out-of-control drinking |
| PSP | 1.00 | .45* | .34* | .06 | .30* |
| AUDIT | .45* | 1.00 | .38* | .24* | .65* |
| Craving rating^ | .34* | .38* | 1.00 | .18 | .31 |
| Years of drinking | .06 | .24* | .18 | 1.00 | .21* |
| Out-of-control drinking | .30* | .65* | .31 | .21* | 1.00 |
|  |  |  |  |  |  |

Note: * significant at the corrected *p* < .005 (Bonferroni). **^** data from 71 subjects who performed the alcohol cue reactivity task.

**1.2 Alcohol cue reactivity (ACR) task**

Following the 10-minute resting-state run with eyes closed, a subsample of 71 subjects (34 females, age M ± SD = 36.1 ± 14.0 years) also performed the alcohol cue reactivity task in the same fMRI session. The remaining 109 subjects (AUDIT = 4.4 ± 3.6, PSP = 21.7 ± 5.6) did not perform the task as they were part of a different study. The 71 subjects who completed the task showed greater AUDIT (10.4 ± 9.9), PSP (21.7 ± 5.6) scores, and out-of-control drinking compared to those who did not (AUDIT = 4.4 ± 3.6, PSP = 18.2 ± 5.7) (independent-samples t-tests *p*’s < .001). The two groups did not significantly differ in age (*p* = .20), years of drinking (*p* = .53), or gender distribution (female percentage: 71-subject group = 46%, 109-subject group = 44%).

The task was previously used and validated in our past studies^3,4^. Participants viewed alternating blocks of alcohol-related pictures (e.g., alcoholic drinks, bar scenes, etc.) and neutral (i.e. non-alcoholic) images (e.g., milk, orange juice, etc.) (**Fig. S1**). In each block, after a 2-s fixation, six pictures displaying alcohol (alcohol block) or neutral (neutral block) cues were shown for 6 s each. Participants were instructed to view the stimuli and contemplate how they may relate to them. At the end of the block, participants were instructed to rate their alcohol craving by using button press. Participants completed two 9-minute runs with each consisting of 6 alcohol and 6 neutral blocks. Participants reported greater craving (on the scale of 0-10, 0 = no craving at all, 10 = highest craving possible) during the alcohol blocks (3.2 ± 2.7) than neutral blocks (2.0 ± 2.1) (*p* < .001). Alcohol craving ratings during the alcohol blocks which showed significant correlations with AUDIT (*r* = .38, *p* = .001) and PSP (*r* = .34, *p* = .004) scores were used in subsequent analyses.


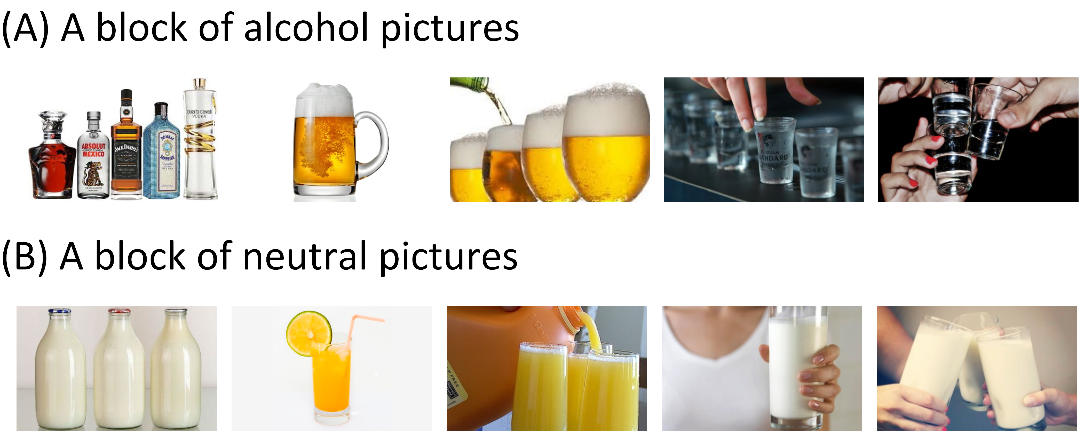


**Figure S1**. Alcohol cue exposure – task schematic. Participants performed 2 runs, each containing 6 blocks of alcohol cues (**A**) and 6 blocks of neutral cues (**B**). Each picture was presented for 6 seconds. Blocks began with a 2-second fixation. Participants were instructed to report their alcohol craving at the end of every block. Each block lasted approximately 45 seconds, including the time for craving rating.

**1.3 Imaging data preprocessing**

Data were analyzed with SPM12 (Wellcome Trust Centre for Neuroimaging). Images from the first five TRs at the beginning of each run were discarded to ensure steady-state equilibrium between RF pulsing and relaxation. Images were then first realigned and corrected for slice timing. A mean functional image volume was constructed for each subject per run from the realigned image volumes. These mean images were co-registered with the high-resolution structural image and segmented for normalization with affine registration, followed by nonlinear transformation with 2.5-mm isotropic voxel size. The normalization parameters determined for the structure volume were then applied to the corresponding functional image volumes for each subject. The images were smoothed with a Gaussian kernel of 4-mm FWHM.

For resting-state functional connectivity (rsFC), we reduced spurious BOLD variances with additional preprocessing. Signals from the ventricles, white matter, and whole brain were removed through a linear regression in addition to the six parameters obtained by rigid body head-motion correction. As BOLD fluctuations below a frequency of 0.1 Hz may contribute to regionally specific BOLD correlations, we applied a temporal band-pass filter (0.009 Hz < ƒ < 0.08 Hz) to the time course to obtain low-frequency fluctuations.

To minimize the effects of micro head motion (>0.1 mm) which represents a significant source of spurious correlations in rsFC analysis, we implemented the “scrubbing” method ^5^ to remove time points affected by head motions. Briefly, for every time point *t*, we computed the frame-wise displacement given by FD (*t*) = | ∆d_x_ (*t*) | + | ∆d_y_ (*t*) | + | ∆d_z_ (*t*) | + | ∆α (*t*) | + | ∆β (*t*) | + | ∆γ (*t*) | where (d_x_, d_y_, d_z_) and (α, β, γ) are the translational and rotational movements, respectively. The second head movement metric was the root mean square variance (DVARS) of the differences in % BOLD intensity I(*t*) between consecutive time points across voxels, computed as follows: DVARS (*t*) = $\sqrt{\langle\left| I \left( t \right)-I \left( t-1 \right) \right|^{2}\rangle}$, where the brackets indicate the mean across voxels. Finally, to compute each subject’s correlation map, we removed time points that exceeded the head motion limit FD (*t*) > 0.5mm or DVARS (*t*) >0.5% ^5^. On average, 1% of the time points were removed across subjects.

**1.4 Analyses of resting-state functional connectivity**

The medial orbitofrontal cortex (mOFC) and periaqueductal gray (PAG) (**Fig. S2**) were chosen as seed for functional connectivity analysis. The mOFC mask was obtained from an imaging meta-analysis examining reward processing (contrast positive > negative effects of subjective value)^6^ and the PAG from the Harvard Ascending Arousal Network^7^. For each subject, the correlation coefficient between the averaged time course of the seed region and that of every other voxel was computed. Correlation maps were then Fisher’s z transformed. The Z maps were used in group, random effect analyses in which we conducted whole-brain multiple regressions against the PSP scores with age and sex as the covariates. As AUDIT scores did not exhibit a normal distribution (*p* = .001, Shapiro Shapiro-Wilk test), we applied log transformation which lessened the skewness but did not normalize the distribution. The log transformed AUDIT score was used in subsequent analyses. The results of the whole-brain multiple regressions were examined with the threshold of p<0.001 (uncorrected) at the voxel level in combination with a threshold of p<0.05, corrected for family-wise error, at the cluster level, according to current reporting standards^8^. All coordinates were reported in MNI space.


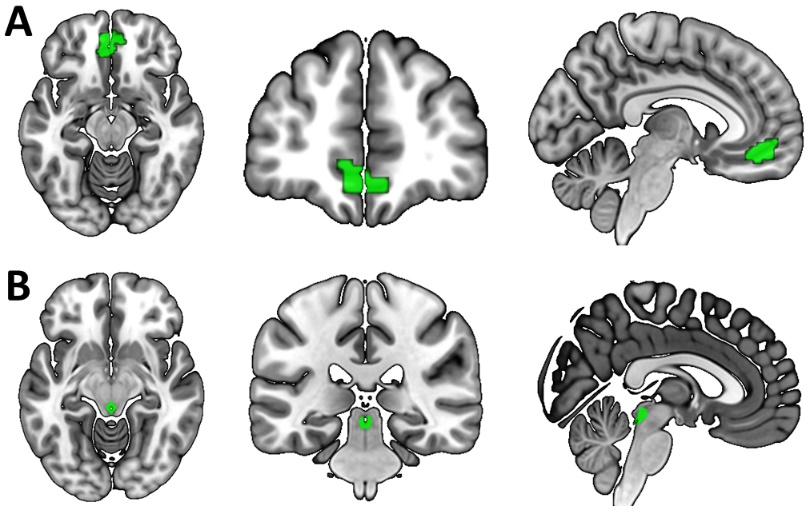


**Figure S2**. The medial orbitofrontal cortex (mOFC, **A**) and periaqueductal gray (PAG, **B**) were chosen as seed for resting-state and cue-elicited functional connectivity analyses.

**1.5 Alcohol cue reactivity and functional connectivities**

As with previous work with similar task designs^9,10^, we distinguished alcohol and neutral blocks for each subject using a general linear model (GLM) which included the realignment parameters in all six dimensions. We corrected for serial autocorrelation caused by aliased cardiovascular and respiratory effects with the FAST model. The GLM was used to estimate the component of variance explained by each regressor. We constructed for every subject contrasts of alcohol vs. neutral blocks to evaluate regional activities that differentiated viewing of alcohol and neutral cues. The contrast images of the subject-level analysis were used for group-level statistical (random-effects) analyses. One-sample t-tests were conducted to identify regional responses to alcohol vs. neutral cues across subjects, using the same threshold as reported above.

The task-state connectivity of the PAG and mOFC during alcohol vs. neutral cues was estimated using general psychophysiological interactions (gPPI) via the gPPI toolbox^11^. A gPPI model was created for each subject, using normalized and smoothed functional EPIs, with three components: the physiological term which represents the time series from the seed region, the psychological term which represents the task conditions (e.g., alcohol, neutral cues), and the psychophysiological interaction term. The gPPI was computed as the element-by-element product of the deconvolved time series of the seed region and a task condition vector^12^. Relevant cue conditions (alcohol, neutral cues) for each block were included in the model. The subject-wise models also included regressors for the onset of all blocks. We used the same PAG and mOFC seeds as in the rsFC analysis and the two cue conditions were included in the model. Condition-specific connectivity for each subject was calculated by contrasting the alcohol and neutral cue blocks as in our previous work^13^.

As the occipital cortex (OC) showed preferential responses to alcohol cues (see *Results*), we examined its gPPI connectivity with the PAG and mOFC. In a region-of-interest analysis, parameter estimates (β weight) of the PAG-OC and mOFC-OC cue-elicited connectivities were extracted for the contrast alcohol > neutral cues. The parameter estimates represented the connectivity strength between two given regions and were examined in relation to PSP scores.

**1.7 Mediation analysis**

To examine the inter-relationships of PAG/mOFC connectivities (see *Results*), PSP, and AUDIT scores, we conducted mediation analyses^14^. The methods were detailed in our previous work ^15,16^. Briefly, in a mediation analysis, the relation between the independent variable X and dependent variable Y (i.e., X → Y) is tested to determine whether it is significantly mediated by a variable M. The mediation test is performed using the following three regression equations:

Y = i1 + cX + e1

Y = i2 + c'X + bM + e2

M = i3 + aX + e3

where *a* represents X → M, *b* represents M → Y (controlling for X), *c'* represents X → Y (controlling for M), and *c* represents X → Y. *a*, *b*, *c*, and *c'* are path coefficients. Variable M is considered a mediator of connection X → Y if (*c* – *c*') is significantly different from zero^14^. If (*c* – *c'*) is different from zero and the paths *a* and *b* are significant, then X → Y is mediated by M. In addition, if path *c'* is not significant, there is no direct connection from X to Y and that X → Y is completely mediated by M. The analysis was performed with package Lavaan^17^ in R (<https://www.r-project.org>). To test the significance of the mediation effect, we used the bootstrapping method as it is generally considered advantageous to the Sobel test^14^.

Specifically, we evaluated the inter-relationships between functional connectivity, PSP, and AUDIT scores, each for PAG and mOFC connectivities (see *Results*). For each seed, we considered all six possible models. To correct for multiple model testing, we applied Bonferroni correction (p ≤ .008).

**1.8 Path analysis**

We evaluated the effects of PAG and mOFC connectivities/activities on PSP scores, AUDIT scores, and alcohol craving ratings. Path analysis^18,19^ involves a set of exogenous variables whose variance is not accounted for by the model and endogenous variables whose variance is explained in part by other variables in the model. Path analysis is conducted with regression analysis which predicts the effects on each endogenous variable from all other variables. The beta weights (β) from these multiple regressions are the path coefficients. Standardized path coefficients convey assumptions about the directionality of interactions between variables. Model fit was assessed with fit indices which included the Root Mean Square Estimation of Approximation (RMSEA, < 0.08 for an acceptable fit), Chi-square (χ^2^/df, < 3), Comparative Fit Index (CFI, >.9), and Standardized Root Mean Square Residual (SRMR, < .06)^20,21^.

Specifically, in the first analysis using resting-state data, we included the PAG and mOFC connectivities (with the overlap precentral gyrus during resting and with the occipital cortex during alcohol cue exposure – see *Results*) as the exogenous variables and alcohol expectancy PSP and AUDIT scores as the endogenous variables (**Fig. 3B**). In this model, PAG and mOFC connectivities modulated alcohol expectancy which in turn affected problem drinking. We examined both direct and indirect effects with bootstrapping^22^ to determine how connectivities from the pain and reward circuitries may modulate problem drinking and whether this modulation was subjected to any mediation effects. Additionally, we considered an alternative path model in which PSP scores influenced AUDIT scores which in turn modulated mOFC-PrCG and PAG-PrCG connectivities (**Fig. S4**).

In the second analysis using data from the ACR task, we included in the model the difference in the strength of PAG-OC connectivity relative to mOFC-OC connectivity as the exogenous variables. PSP, AUDIT, and alcohol craving ratings represented the endogenous variables. We tested the model in which difference in connectivity strength of PAG/mOFC indirectly modulating problem drinking by influencing alcohol expectancy and craving. Direct and indirect effects were assessed as in the first analysis. An alternative model in which PSP expectancy indirectly modulated PAG/mOFC connectivity strength difference via problem drinking and craving (**Fig. S5**) was also considered.

**2. SUPPLEMENTAL RESULTS**

**2.1 Resting-state results**

**2.1.1 Whole-brain multiple regression analysis with PSP as predictor**

The whole-brain multiple regression of the PAG showed a negative correlation between PSP scores and PAG connectivity with the somatosensory cortex, including the bilateral precentral gyrus (PrCG), right postcentral gyrus, and paracentral lobule (**Table S2**).

The whole-brain multiple regression of the mOFC connectivity showed a positive correlation between PSP scores and mOFC connectivity with the left PrCG (**Table S2**).

| **Table S2**: Resting-state whole-brain regression with PSP as predictor | | | | | | |
| --- | --- | --- | --- | --- | --- | --- |
|  |  | MNI coordinates (mm) | | | Voxel | Cluster |
|  | Region | x | y | z | T | k |
| **PAG connectivity** | |  |  |  |  |  |
|  | Paracentral lobule | -8 | -28 | 66 | 5.22 | 172 |
|  |  | 5 | -21 | 63 | 4.14 |  |
|  | Precentral gyrus | -28 | -28 | 63 | 4.01 |  |
|  |  | 40 | -8 | 46 | 4.37 | 48 |
|  |  | -38 | -18 | 58 | 4.41 | 45 |
|  | Postcentral gyrus | 30 | -31 | 58 | 4.24 | 70 |
| **mOFC connectivity** | |  |  |  |  |  |
|  | Precentral gyrus | -42 | -31 | 50 | 5.30 | 235 |
|  |  | -55 | -14 | 33 | 4.16 |  |
|  |  | -45 | -21 | 40 | 3.88 |  |
|  |  |  |  |  |  |  |

*All activation significant at p voxel < .001 (uncorrected) and p cluster < .05 (FWE)*

**2.1.2 Mediation analysis**

*PAG connectivity, PSP, and AUDIT scores*

Mediation analysis for PAG connectivity, PSP, and AUDIT scores. PAG connectivity is the averaged parameter estimates of PAG with the paracentral lobule, postcentral gyrus, and PrCG defined from the multiple regression against PSP. Results showed two models (Model 1 and Model 2) with significant mediation effects after correction for multiple model testing (**Table S3**). Specifically, in Model 1, PAG connectivity (independent variable) reduced PSP expectancy (mediator), which in turn decreased problem drinking (dependent variable): PAG connectivity → PSP → AUDIT. The model showed a significant mediation effect after correction for multiple model testing (*c* - *c*' = -2.36, *p* < .001). The path coefficient *c* (i.e., PAG connectivity → AUDIT before accounting for the mediating effect of PSP) was significant (*p* = .006) and the path coefficient *c*' (i.e., after accounting for the mediating effect) was not significant (*p* = .615). Thus, PSP expectancy fully mediated the relationship between PAG connectivity and problem drinking. Model 2 (AUDIT → PSP → PAG connectivity) also showed a significant mediation effect (*c* - *c*' = -.017, *p* < .001). Alcohol expectancy fully mediated the relationship between problem drinking and PAG connectivity. None of the other four models was significant (corrected *p*’s > .06). Thus, enhanced PAG connectivity was associated with lower drinking severity and this negative relationship was bidirectionally mediated by reduced expectancy of PSP.

| **Table S3**. Mediation of PAG connectivity, PSP, and AUDIT | | | | | |
| --- | --- | --- | --- | --- | --- |
|  | Path *a* (*X* → *M*) | Path b (*M* → *Y*) | Path *c* (*X* → *Y*) | Path *c*' (*X* → *Y*) | Mediation path (*c* - *c*') |
| Model 1: *X* (PAG connectivity) → *Y* (AUDIT) mediated by *M* (PSP) | | | | | |
| β | -31.80 | 0.07 | -1.96 | 0.40 | -2.36 |
| p-values | 0.000 | 0.000 | 0.006 | 0.615 | 0.001 |
| CI | -39.81 -23.97 | 0.04 0.10 | -3.42 -0.59 | -1.15 1.99 | -3.39 -1.40 |
| Model 2: *X* (AUDIT) → *Y* (PAG connectivity) mediated by *M* (PSP) | | | | | |
| β | 2.80 | -0.01 | -0.01 | 0.00 | -0.02 |
| p-values | 0.000 | 0.000 | 0.013 | 0.611 | 0.000 |
| CI | 1.808 3.588 | -0.01 -0.004 | -0.025 -0.004 | -0.009 0.014 | -0.027 -0.010 |
| Model 3: *X* (AUDIT) → *Y* (PSP) mediated by *M* (PAG connectivity) | | | | | |
| β | -0.01 | -27.09 | 2.80 | 2.42 | 0.38 |
| p-values | 0.013 | 0.000 | 0.000 | 0.000 | 0.011 |
| CI | -0.025 -0.004 | -36.05 -18.26 | 1.808 3.588 | 1.420 3.231 | 0.124 0.705 |
| Model 4: *X* (PSP) → *Y* (AUDIT) mediated by *M* (PAG connectivity) | | | | | |
| β | -0.01 | 0.40 | 0.07 | 0.07 | 0.00 |
| p-values | 0.000 | 0.615 | 0.000 | 0.000 | 0.614 |
| CI | -0.008 -0.004 | -1.150 1.99 | 0.044 0.099 | 0.043 0.10 | -0.012 0.007 |
| Model 5: *X* (PAG connectivity) → *Y* (PSP) mediated by *M* (AUDIT) | | | | | |
| β | -1.96 | 2.42 | -31.83 | 27.09 | -4.74 |
| p-values | 0.006 | 0.000 | 0.000 | 0.000 | 0.02 |
| CI | -3.423 -0.59 | 1.420 3.23 | -39.8 -23.97 | 36.054 -18.26 | -9.671 -1.42 |
| Model 6: *X* (PSP) → *Y* (PAG connectivity) mediated by *M* (AUDIT) | | | | | |
| β | 0.072 | 0.003 | -0.006 | -0.006 | 0 |
| p-values | 0.000 | 0.611 | 0.000 | 0.000 | 0.628 |
| CI | 0.044 0.09 | -0.009 0.01 | -0.008 -0.004 | -0.008 -0.004 | -0.001 0.001 |
|  |  |  |  |  |  |

*Medial OFC connectivity, PSP, and AUDIT scores*

Mediation analysis showed two models (Model 1 and Model 2) with significant mediation effects after correction for multiple model testing (**Table S4**). Thus, elevated mOFC connectivity was associated with increased drinking severity and this positive relationship was bidirectionally mediated by heightened expectancy of PSP.

| **Table S4**. Mediation of mOFC connectivity, PSP, and AUDIT | | | | | |
| --- | --- | --- | --- | --- | --- |
|  | Path *a* (*X* → *M*) | Path b (*M* → *Y*) | Path *c* (*X* → *Y*) | Path *c*' (*X* → *Y*) | Mediation path (*c* - *c*') |
| Model 1: *X* (mOFC connectivity) → *Y* (AUDIT) mediated by *M* (PSP) | | | | | |
| β | 24.41 | 0.067 | 1.20 | -0.44 | 1.64 |
| p-values | 0.000 | 0.000 | 0.101 | 0.550 | 0.000 |
| CI | 16.044 33.035 | 0.035 0.09 | -0.184 2.69 | -1.793 1.084 | 0.863 2.655 |
| Model 2: *X* (AUDIT) → *Y* (mOFC connectivity) mediated by *M* (PSP) | | | | | |
| β | 2.52 | 0.008 | 0.01 | -0.01 | 0.02 |
| p-values | 0.000 | 0.000 | 0.070 | 0.564 | 0.001 |
| CI | 1.485 3.41 | 0.005 0.011 | -0.002 0.03 | -0.024 0.01 | .010 0.033 |
| Model 3: *X* (AUDIT) → *Y* (PSP) mediated by *M* (mOFC connectivity) | | | | | |
| β | 0.01 | 21.76 | 2.52 | 2.21 | 0.31 |
| p-values | 0.078 | 0.000 | 0.000 | 0.000 | 0.104 |
| CI | -0.002 0.03 | 14.045 29.74 | -0.024 0.733 | 1.218 3.08 | -0.024 0.733 |
| Model 4: *X* (PSP) → *Y* (AUDIT) mediated by *M* (mOFC connectivity) | | | | | |
| β | 0.01 | -0.44 | 0.06 | 0.07 | 0.00 |
| p-values | 0.000 | 0.55 | 0.000 | 0.000 | 0.557 |
| CI | 0.005 0.010 | -1.793 1.08 | 0.037 0.09 | 0.035 0.096 | -0.014 0.007 |
| Model 5: *X* (mOFC connectivity) → *Y* (PSP) mediated by *M* (AUDIT) | | | | | |
| β | 1.20 | 2.21 | 24.41 | 21.76 | 2.66 |
| p-values | 0.101 | 0.000 | 0.000 | 0.000 | 0.126 |
| CI | -0.184 2.698 | 1.218 3.081 | 16.044 33.03 | 14.045 29.74 | -0.176 6.95 |
| Model 6: *X* (PSP) → *Y* (mOFC connectivity) mediated by *M* (AUDIT) | | | | | |
| β | 0.064 | -0.005 | 0.007 | 0.008 | 0 |
| p-values | 0.000 | 0.564 | 0.000 | 0.000 | 0.590 |
| CI | 0.037 0.09 | -0.024 0.01 | 0.005 0.01 | 0.005 0.011 | -0.002 0.001 |
|  |  |  |  |  |  |

**2.1.3 PAG and mOFC connectivity with the shared substrate PrCG**

The resting-state connectivity maps of PAG and mOFC overlapped in the left PrCG. PAG-PrCG connectivity negatively predicted PSP scores whereas mOFC-PrCG connectivity positively predicted PSP scores (**Fig. S3).**


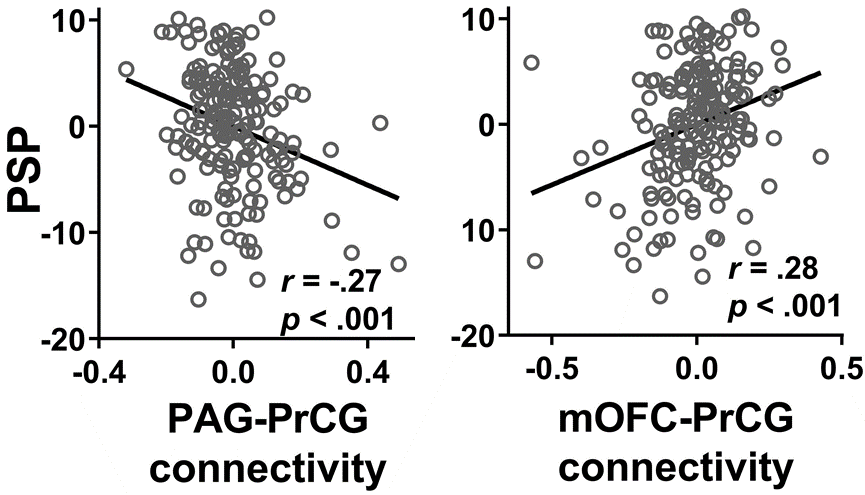


**Figure S3**. The PAG resting-state connectivity with the overlap PrCG negatively predicted PSP scores (left) whereas the mOFC resting-state connectivity with the overlap PrCG positively predicted PSP scores (right).

**2.1.4 Path analysis – alternative model for resting-state data**

The alternative model in which PSP scores influenced AUDIT which in turn modulated PAG and mOFC connectivities showed a poor fit (Fit indices: RMSEA = .23, χ^2^/df = 10.80, SRMR = .10, and CFI = .73, **Fig. S4A**) and was not considered further.


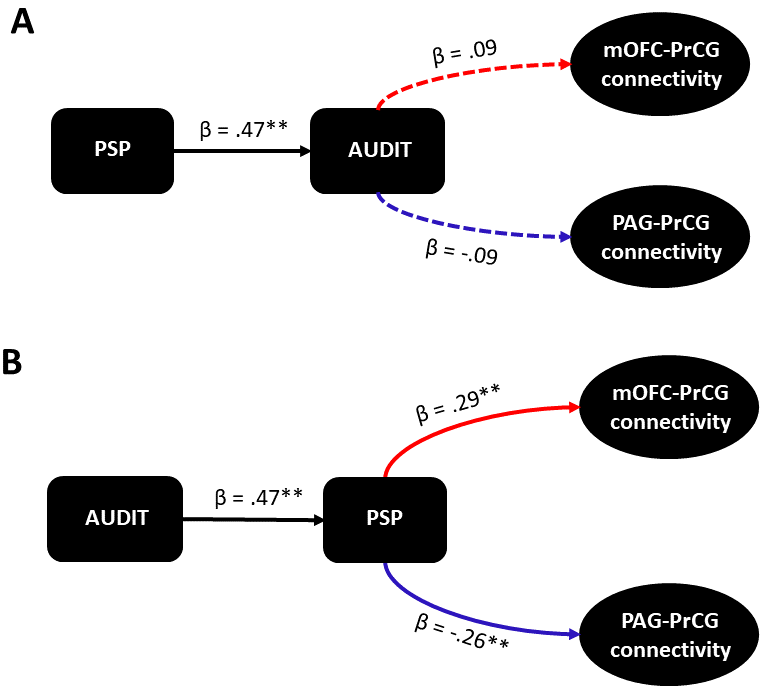


**Figure S4**. (**A**) The alternative path model in which PSP indirectly influenced PAG and mOFC connectivities via AUDIT showed an inferior fit. (**B**) The model in which expectancy of drinking pleasure modulated PAG and mOFC connectivity via drinking severity exhibited a good fit. Solid and dotted lines indicate significant and non-significant paths, respectively.

To determine whether drinking severity modulated connectivity, we evaluated another model in which PSP influenced PAG and mOFC connectivity via AUDIT scores (**Fig. S4B**). The model showed a good fit (Fit indices: RMSEA = 0.00, χ^2^/df = .245, SRMR = .333, and CFI = 1.00).

**2.2 Alcohol cue reactivity task results**

**2.2.1 Cue-elicited activations**

One-sample t-tests showed significant activations to alcohol vs. neutral cues in the occipital cortex, mOFC, posterior cingulate cortex, and superior frontal gyrus (**Table S5**). The reversed contrast showed significant activation in the parieto-occipital sulcus.

| **Table S5**: Alcohol cue reactivity task | | | | | | |
| --- | --- | --- | --- | --- | --- | --- |
|  |  | MNI coordinates (mm) | | | Voxel | Cluster |
|  | Region | x | y | z | T | k |
| **Alcohol > Neutral** | |  |  |  |  |  |
|  | Occipital cortex | -20 | -96 | 3 | 9.44 | 2415 |
|  |  | 28 | -94 | 13 | 8.82 |  |
|  |  | 15 | -96 | 3 | 8.54 |  |
|  | mOFC | -8 | 39 | -14 | 6.52 | 292 |
|  |  | -12 | 44 | -10 | 5.35 |  |
|  |  | -10 | 52 | -7 | 5.22 |  |
|  | PCC | -5 | -54 | 20 | 5.66 | 141 |
|  |  | -2 | -56 | 13 | 5.01 |  |
|  |  | 8 | -48 | 23 | 4.40 |  |
|  | SFG | -20 | 32 | 43 | 5.30 | 324 |
|  |  | -12 | 39 | 46 | 5.14 |  |
|  |  | -12 | 46 | 33 | 4.84 |  |
| **Neutral > Alcohol** | |  |  |  |  |  |
|  | Parieto-occipital sulcus | 12 | -81 | 36 | 4.90 | 181 |
|  |  | 8 | -74 | 33 | 4.46 |  |
|  |  | -5 | -84 | 33 | 4.15 |  |
|  |  |  |  |  |  |  |

*Abbreviations: mOFC – medial orbitofrontal cortex, PCC – posterior cingulate cortex, SFG – superior frontal gyrus. All activations significant at p voxel < .001 (uncorrected) and p cluster < .05 (FWE).*

**2.2.2 Path analysis**

The alternative model in which PSP scores indirectly influenced PAG/mOFC connectivity difference via AUDIT and craving showed a poor fit (Fit indices: RMSEA = .29, χ^2^/df = 6.92, SRMR = .08, and CFI = .81, ***Fig. S5A***) and was not considered further.


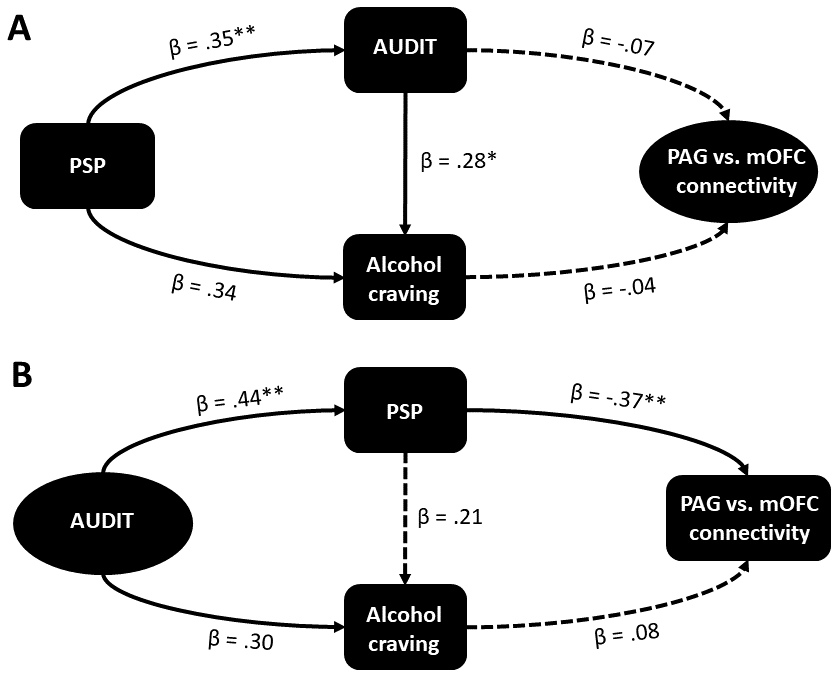


**Figure S5**. (**A**) The alternative path model in which PSP expectancy indirectly influenced PAG/mOFC connectivity difference via problem drinking and craving showed an inferior fit. (**B**) The model in which expectancy of drinking pleasure modulated OC connectivity via drinking severity exhibited a good fit. Solid and dotted lines indicate significant and non-significant paths, respectively.

As with the resting state analysis, we evaluated the path model in which expectancy of drinking pleasure indirectly modulated the connectivity difference between OC and PAG vs. OC and mOFC via drinking severity showed a good fit (Fit indices: RMSEA = 0.00, χ^2^/df = .245, SRMR = .015, and CFI = 1.00). Thus, the relationship between drinking severity and OC connectivity is bidirectional.

**2.2.3 Alternative definition of the OC**

To rule out the possibility that the OC activation had overlap with the white matter we restricted the voxels to 1) a more stringent threshold (voxel-level *p* < .05, FWE) of the contrast alcohol > neutral cues, and 2) the group’s averaged gray matter segmentation image (**Fig. S6A**). The restriction yielded an OC cluster with 854 voxels (compared to the 2,415 voxels in our original report). Using this restricted OC, we repeated our analyses and found almost identical findings. Specifically, the alcohol cue-elicited OC connectivity strength between PAG and mOFC (i.e., PAG connectivity with OC minus mOFC connectivity with OC during alcohol > neutral cues) was negatively correlated with PSP scores (*r* = .01, *p* = .30, Fig. S6B).


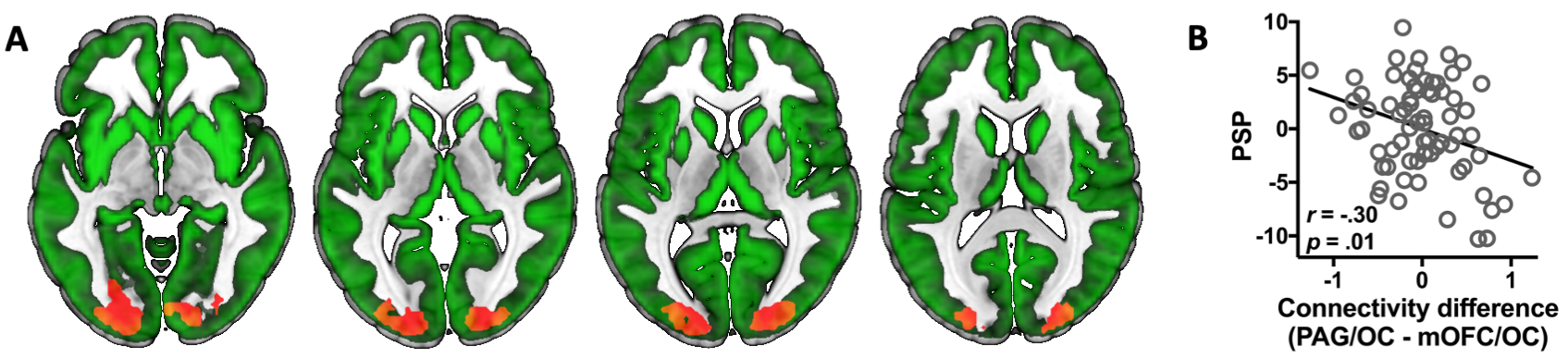


**Figure S6**: We subjected the OC (red) to restriction by the group gray matter mask (green) to ensure the seed does not include white matter (A). Using this OC, we calculated the connectivity strength difference for the OC with the PAG vs. the mOFC and found that this connectivity strength exhibited a negative relationship with PSP scores (B), as previously reported in our main text.

Similarly, the path analysis in which PAG/mOFC connectivity strength difference indirectly influenced AUDIT scores by negatively modulating PSP and alcohol craving also showed a model with good fit. Briefly, the model showed a good fit (Fit indices: RMSEA = 0.00 [90% CI: 0.00 .25], χ^2^/df = 0.25, SRMR = .015, and CFI = 1.00). The alternative model in which PSP expectancy indirectly influenced PAG/mOFC connectivity difference via problem drinking and craving showed a poor fit (Fit indices: RMSEA = .34, χ^2^/df = 9.23, SRMR = .1, and CFI = .76). Thus, using a more stringent definition of the OC did not materially change our results or their interpretation.

**References**

1 George WH, Frone MR, Cooper ML, Russell M, Skinner JB, Windle M. A revised Alcohol Expectancy Questionnaire: factor structure confirmation, and invariance in a general population sample. *J Stud Alcohol* 1995; **56**: 177–185.

2 Saunders J, Assland O, Babor T, De la fuente J, Grant M. Development of the alcohol use disorders identification test (AUDIT): WHO collaborative project on early detection of persons with harmful alcohol consumption-II. *Addiction* 1993; **88**: 701–904.

3 Wang W, Zhornitsky S, Le TM, Dhingra I, Zhang S, Krystal JH *et al.* Cue-elicited craving, thalamic activity, and physiological arousal in adult non-dependent drinkers. *J Psychiatr Res* 2019; **116**: 74–82.

4 Wang W, Zhornitsky S, Le TM, Zhang S, Li CR. Heart rate variability, cue-evoked ventromedial prefrontal cortical response, and problem alcohol use in adult drinkers. *Biol Psychiatry Cogn Neurosci Neuroimaging* 2019. doi:10.1016/j.bpsc.2019.12.013.

5 Power JD, Barnes KA, Snyder AZ, Schlaggar BL, Petersen SE. Spurious but systematic correlations in functional connectivity MRI networks arise from subject motion. *Neuroimage* 2012; **59**: 2142–2154.

6 Bartra O, McGuire JT, Kable JW. The valuation system: A coordinate-based meta-analysis of BOLD fMRI experiments examining neural correlates of subjective value. *Neuroimage* 2013; **76**: 412–427.

7 Edlow BL, Takahashi E, Wu O, Benner T, Dai G, Bu L *et al.* Neuroanatomic connectivity of the human ascending arousal system critical to consciousness and its disorders. *J Neuropathol Exp Neurol* 2012; **71**: 531–546.

8 Eklund A, Nichols TE, Knutsson H. Cluster failure: Why fMRI inferences for spatial extent have inflated false-positive rates. *Proc Natl Acad Sci* 2016; **113**: 7900–7905.

9 Zhornitsky S, Zhang S, Ide JS, Chao HH, Wang W, Le TM *et al.* Alcohol Expectancy and Cerebral Responses to Cue-Elicited Craving in Adult Nondependent Drinkers. *Biol Psychiatry Cogn Neurosci Neuroimaging* 2018; : 1–12.

10 Le TM, Zhornitsky S, Wang W, Zhang S, Li CR. Problem drinking alters gray matter volume and food cue responses of the lateral orbitofrontal cortex. *Addict Biol* 2019; : 1–10.

11 McLaren DG, Ries ML, Xu G, Johnson SC. A generalized form of context-dependent psychophysiological interactions (gPPI): A comparison to standard approaches. *Neuroimage* 2012; **61**: 1277–1286.

12 Garraux G. Shared Brain Areas But Not Functional Connections Controlling Movement Timing and Order. *J Neurosci* 2005; **25**: 5290–5297.

13 Le TMTM, Borghi JAJA, Kujawa AJAJ, Klein DN, Leung H-CH-C. Alterations in visual cortical activation and connectivity with prefrontal cortex during working memory updating in major depressive disorder. *NeuroImage Clin* 2017; **14**: 43–53.

14 MacKinnon DP, Fairchild AJ, Fritz MS. Mediation Analysis. *Annu Rev Psychol* 2007; **58**: 593–614.

15 Le TM, Wang W, Zhornitsky S, Dhingra I, Zhang S, Li C-SR. Interdependent Neural Correlates of Reward and Punishment Sensitivity During Rewarded Action and Inhibition of Action. *Cereb Cortex* 2019; : 1–15.

16 Le TM, Zhornitsky S, Wang W, Ide J, Zhang S, Li C-SR. Posterior cingulate cortical response to active avoidance mediates the relationship between punishment sensitivity and problem drinking. *J Neurosci* 2019; **39**: 0508–19.

17 Rosseel Y. Lavaan: an R package for structural equation modeling and more. *J Stat Softw* 2012; **48**: 1–36.

18 Duncan OD. Path Analysis: Sociological Examples. *Am J Sociol* 1966; **72**: 1–16.

19 Wuensch KL. Introduction to Path Analysis. In: *Introduction to Path Analysis*. 2016, pp 1–18.

20 Hu L-T, Bentler PM. Evaluating model fit. In: *Structural equation modeling: Concepts, issues, and applications.* Sage Publications, Inc: Thousand Oaks, CA, US, 1995, pp 76–99.

21 Chen F, Curran PJ, Bollen KA, Kirby J, Paxton P. An empirical evaluation of the use of fixed cutoff points in RMSEA test statistic in structural equation models. *Sociol Methods Res* 2008; **36**: 462–494.

22 Mahmoud AB, Grigoriou N. When empathy hurts: Modelling university students’ word of mouth behaviour in public vs. private universities in Syria. *High Educ Q* 2017; **71**: 369–383.
